# Outdoor Microphone Range Tests and Spectral Analysis of UAV Acoustic Signatures for Array Development

**DOI:** 10.3390/s25227057

**Published:** 2025-11-19

**Authors:** Gabriel Jekateryńczuk, Zbigniew Piotrowski

**Affiliations:** Faculty of Electronics, Military University of Technology, 00-908 Warsaw, Poland; zbigniew.piotrowski@wat.edu.pl

**Keywords:** microphone array, unmanned aerial vehicle, outdoor field measurements, acoustic UAV detection, spectral analysis

## Abstract

Acoustic sensing is a passive and cost-effective option for unmanned aerial vehicle detection, where both signal processing and microphone hardware jointly determine field performance. In this study, we focus on the hardware front-end as a foundation for improving the reliability of subsequent DSP- or AI-based detection methods. We present a detection-focused comparison of several microphones in outdoor tests, combining calibrated range measurements with spectral analysis of real unmanned aerial vehicle emissions from three platforms. We report hardware metrics only: signal-to-noise ratio, effective detection range, attenuation slope with distance, and the low-frequency background floor. Across wind conditions and source orientations, the RØDE NTG-2 with WS6 windshield delivered the most balanced performance: in strong wind, it extended the detection range over the bare NTG-2 by approximately 31–131% (depending on azimuth), lowered the low-frequency noise floor by about 2–3 decibels, and matched or increased the wideband signal-to-noise ratio by 1.8–4.4 decibels. A parabolic NTG-2 achieved very low background noise levels at low frequencies and strong on-axis reach but proved vulnerable to gust-induced transients. Based on this evidence, we propose an eight-channel, dual-tier array of NTG-2 + WS6 elements that preserves near-hemispherical coverage and phase coherence, establishing a practical hardware baseline for outdoor acoustic unmanned aerial vehicle detection and a reproducible platform for subsequent localization and classification studies.

## 1. Introduction

In recent years, unmanned aerial vehicles (UAVs) have become widely adopted across both civilian and military sectors [1]. Their ability to perform autonomous or remote-controlled missions with high flexibility has led to a wide range of practical applications. Civilian use cases include infrastructure inspection, precision agriculture, mapping, and logistics [2]. In the defense sector, UAVs play a vital role in surveillance, reconnaissance, and tactical support [3].

As drone technology matures, platforms are becoming smaller and quieter, thanks to advances in battery systems, lightweight materials, and miniaturized electronics [4]. At the same time, advances in autonomy and communication systems have made UAVs increasingly capable of operating beyond the visual line of sight (BVLOS). These advancements have significantly expanded the operational envelope of UAVs, while also raising new challenges for airspace management, public safety, and the protection of critical infrastructure. In response, regulatory frameworks have begun to evolve, introducing operational limits, mandatory registration, and flight authorization systems [5,6,7]. However, the rapid pace of UAV adoption has outpaced enforcement capabilities in many regions.

Beyond legitimate uses, the same technological improvements have enabled malicious or unauthorized applications—including smuggling, contraband delivery into prisons, espionage, and disruption of airport operations [8]. In conflict zones, UAVs are increasingly deployed for reconnaissance and precision attacks by both state and non-state actors [9]. These risks highlight the urgent need for effective and scalable drone detection systems that can operate in diverse environments.

The increasing presence of UAVs has driven the development of various detection technologies. A wide variety of sensing modalities have been explored for this purpose, including radar [10], electro-optical and infrared imaging [11], radio frequency (RF) [12], and acoustics [13]. Each approach presents distinct advantages and limitations. Radar and RF systems can detect UAVs at long distances but are often limited by electromagnetic clutter, terrain masking, or regulatory constraints. Optical systems offer high-resolution tracking but require a line of sight and are sensitive to weather and lighting conditions. In contrast, acoustic detection offers a passive and cost-effective solution capable of identifying both manually controlled and autonomous UAVs. It operates independently of the drone’s communication link or visibility and can detect drones solely by their characteristic acoustic signatures.

Given the limitations of each detection method, research has increasingly focused on combining them through multi-sensor fusion. Recent studies have demonstrated the effectiveness of such integrations, including RF–acoustic fusion drone classification in low signal-to-noise ratio (SNR) environments [14], radar–acoustic systems for improved detection under outdoor noise conditions [15], or audio–visual approaches for trajectory estimation and tracking in complex visual scenes [16].

While sensor fusion improves robustness at the system level, each sensing modality still relies on effective signal processing within its own domain. In acoustics, detection performance depends strongly on how UAV sound signatures are captured and analyzed. As a result, a substantial body of research has explored digital signal processing (DSP) techniques for acoustic event detection, localization, and tracking. Early work on sound event detection demonstrated that temporal and spectral features extracted from microphone networks can be used to detect, classify, and monitor acoustic events in complex soundscapes [17,18].

Within UAV acoustics, DSP-based methods have been applied not only for sound event detection but also for spatial localization and trajectory estimation of flying platforms. Classical algorithms such as time difference of arrival (TDOA), difference of arrival (DOA), beamforming, and steered response power (SRP-PHAT), as well as subspace approaches like multiple signal classification (MUSIC), have been successfully employed to estimate UAV direction or position under real-world conditions [19]. Beamforming and acoustic camera systems have also been shown to enhance spatial awareness and tracking performance [20,21]. These works confirm the feasibility of acoustic-based UAV detection and localization but primarily emphasize algorithmic signal processing rather than sensor performance.

As computational resources and available data increased, these traditional DSP methods were gradually complemented by statistical and machine-learning-based frameworks. Classical machine learning algorithms such as support vector machines, random forests, and Gaussian mixture models [22] have been used to classify UAV signatures based on handcrafted acoustic features (e.g., Mel-frequency cepstral coefficients or spectral roll-off). These approaches improved generalization and detection robustness under variable noise conditions but still relied on manual feature engineering [23,24].

Building on these developments, deep learning has become a central component of modern sensing and decision-making systems [25]. Deep learning models—particularly convolutional and recurrent neural networks—have demonstrated strong capabilities in extracting meaningful patterns from complex data. They are widely used in tasks such as image recognition, object detection, image analysis, watermarking, and remote sensing, where learning spatial hierarchies and localized features is essential [26,27,28,29,30]. In contrast, recurrent architectures, including LSTM networks, are particularly effective for modeling temporal dynamics in sequential data such as speech or acoustic signals [31,32]. This temporal modeling is especially relevant in UAV acoustics, where the motion and changing acoustic signature over time require time-aware processing for accurate detection, classification, and tracking [25,26].

However, the performance of such AI-based systems is highly dependent on the availability of large, diverse, and well-annotated training datasets. In the context of UAV acoustics, publicly available datasets remain limited in terms of platform variety, environmental diversity, and labeling consistency. Moreover, although several studies propose the use of microphone arrays for UAV detection and localization, they often employ low-cost MEMS microphones without providing systematic justification for their selection or evaluating their performance under real-world, outdoor conditions [33,34,35,36,37,38]. While MEMS microphones offer advantages such as compact size, low power consumption, and very low cost—making large-scale arrays with tens or even hundreds of elements feasible—they generally exhibit lower sensitivity, higher self-noise, and reduced dynamic range compared to professional condenser microphones. Typical MEMS devices provide sensitivities of approximately −40 to −45 dBV/Pa, self-noise levels around 30–35 dBA, and dynamic ranges below 95 dB. These specifications are sufficient for short-range or controlled indoor applications but limit detection capability at greater distances or in acoustically complex environments. In contrast, professional condenser microphones commonly used in acoustic measurement or recording applications generally achieve sensitivities near –34 dBV/Pa, self-noise below 20 dBA, and dynamic ranges exceeding 110 dB. Such differences yield a substantially higher signal-to-noise ratio and improved ability to capture weak UAV emissions over longer ranges.

Furthermore, most existing works do not systematically analyze how microphone performance influences detection accuracy or array design, and condenser-based alternatives have received little attention despite their higher sensitivity and lower intrinsic noise. For this reason, the present study focuses on evaluating higher-sensitivity condenser microphones as potential reference elements for future array development. Previous reviews on multichannel 3D microphone arrays [39] have mainly focused on spatial configuration and beamforming performance for immersive audio capture. In contrast, the present work provides an element-level evaluation of microphone hardware to quantify its impact on long-range acoustic detection under outdoor conditions, thereby directly addressing the performance limitations of MEMS-based systems and providing experimental evidence under realistic field conditions. The choice of microphone hardware—particularly its spectral sensitivity and directional characteristics—directly affects the quality of the data used to train and evaluate AI-based models.

While numerous studies have investigated AI-based acoustic detection and classification of UAVs, the sensing front-end remains the least examined stage of such systems. The existing literature typically reports the use of unspecified or generic MEMS microphones without quantitative comparisons of their detection range, noise performance, or directional response. As a result, the practical acoustic coverage of UAV detection systems is often constrained by sensor limitations rather than algorithmic capability. 

To address this gap, the present work provides an experimental comparison of representative microphone types (shotgun, omnidirectional, large-diaphragm condenser, and parabolic dish) to determine which architectures offer the most effective trade-off between sensitivity, wind-noise immunity, and detection distance. In addition to range-based testing, we perform spectral analysis of UAV emissions, focusing on deterministic tonal components associated with the blade-passing frequency (BPF) and its harmonics [40].

The main contributions of this paper are:

- A detailed experimental evaluation and comparison of multiple microphone configurations for UAV sound capture in outdoor environments with varying wind conditions.
- A comparative analysis of effective recording range, signal-to-noise ratio, and attenuation characteristics using a calibrated narrowband reference signal.
- A spectral characterization of UAV acoustic emissions combined with a systematic assessment of microphone-dependent sensitivity, tonal SNR, and low-frequency noise floor, linking spectral content to acoustic signal prominence in outdoor conditions.
- The design and deployment of a dual-tier, eight-channel microphone array optimized for hemispherical UAV detection based on empirical findings.

The remainder of this paper is organized as follows: Section 2 describes the materials and methods, including the equipment used and the measurement scenarios. Section 3 presents the results of the experiments, covering both the effective recording range and spectral features of UAV emissions. Section 4 describes the design of the developed acoustic array and discusses potential refinements based on field test observations. Section 5 contains a summary of the paper.

## 2. Materials and Methods

To achieve the research objectives, a series of experiments were conducted using various types of microphones and real-world acoustic sources, including test signals and recordings of unmanned aerial vehicles. This section provides a detailed description of the equipment used, measurement scenarios, test setup, and signal analysis methods. The following methodology ensures the reproducibility of the study and forms the basis for interpreting the results presented in the subsequent sections.

### 2.1. Equipment

The equipment used in the experimental setup included a selection of microphones with varying directional and acoustic properties, an audio interface, a recording workstation, and signal playback hardware.

The selection of microphones was intended to encompass devices with diverse directional patterns, sensitivities, and price ranges in order to represent both professional and consumer recording scenarios. The Rode NTG-2, a shotgun condenser microphone, was selected for its high directivity and sensitivity, making it well-suited for capturing focused sound in outdoor environments. The Mozos VMic represents an accessible, low-cost microphone, included to assess the feasibility of affordable solutions for UAV noise acquisition. In contrast, the Behringer B-1 and B-2 Pro large-diaphragm condenser microphones offer studio-grade reference recordings, featuring adjustable polar patterns and low self-noise. This set of microphones thus covers a wide spectrum of acoustic and technical characteristics, enabling comparative analysis. Dedicated measurement microphones were not included, as the study focused on evaluating practical condenser and directional microphones representative of real-world UAV detection scenarios rather than conducting laboratory-grade SPL calibration.

**Figure 1:**
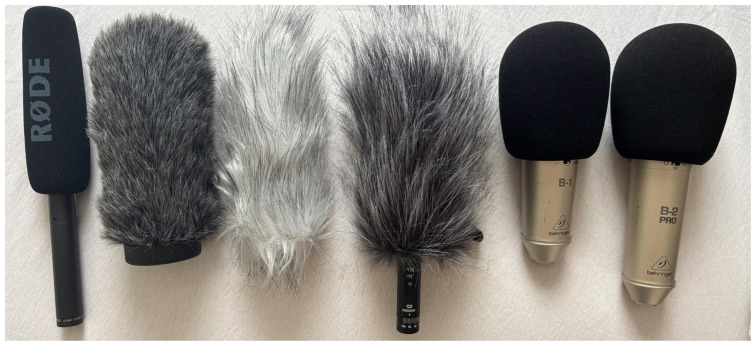
Left to right: RØDE NTG-2 (with foam windscreen), RØDE WS6 (furry windshield), RØDE DeadCat (furry windshield), Mozos VMic (furry windshield), Behringer B-1, Behringer B-2 PRO.

The overall experimental configuration was designed to enable simultaneous, high-resolution recording across multiple channels under repeatable conditions, ensuring that differences in microphone performance could be directly compared. Table 1 summarizes the microphone models evaluated in the experiments, together with their key technical specifications, while Figure 1 illustrates the microphones and the wind-protection configurations used.

All microphones were connected to a Behringer UMC1820 [44] audio interface via balanced XLR cables. The interface was configured with maximum gain for all input channels to ensure consistent and comparable signal levels across devices and to maintain measurable SNR at long distances for small UAVs. This configuration was verified to remain within the preamplifier’s linear operating range for all tested UAVs, producing moderate sound pressure levels (≤70 dB SPL at 1 m). However, this setting may not generalize to higher-thrust or industrial UAVs that generate stronger acoustic emissions or complex propeller interactions (e.g., contra-rotating systems). In such cases, lower gain settings would be required to avoid preamplifier saturation and preserve signal linearity.

Multichannel audio was recorded on a laptop running Ubuntu Linux, using a self-made recording application [45], with a sampling rate of 96 kHz and 24-bit resolution. A BLOW BT460 Bluetooth speaker was used to generate a reference 1 kHz sine wave for range testing. To obtain ground-truth acoustic data, a reference sound pressure level (SPL) was measured using a calibrated sound level meter placed at a known position within the test setup. This measurement served as a baseline for comparing the relative SPL values recorded by the tested microphones at various distances. While the reference SPL measurement does not constitute a full ISO 5305-compliant UAV noise test, it follows the general principles of that standard—namely, the use of a calibrated SPL reference in an open-field configuration—to ensure consistency across microphone comparisons. All source–microphone distances were measured manually using a mechanical distance wheel to ensure reliable ground-level distance tracking during field tests.

Microphones were mounted on a standard microphone stand at a height of approximately 1.5 m above ground level (AGL), with a spacing that minimized mutual acoustic interference. Each microphone was oriented horizontally, i.e., directed with its main axis to the source of the sound. This setup was chosen to maintain consistent angular positioning across all test conditions. The RØDE NTG-2 was additionally tested with an 18-inch parabolic reflector, mounted on a dedicated holder aligned with the microphone’s acoustic axis. Wind protection was evaluated using RØDE WS6 and DeadCat windshields under outdoor conditions to assess their influence on spectral response and effective signal-to-noise ratio.

All recording equipment, including the audio interface and connected microphones requiring phantom power, was powered via a portable battery power station with an integrated inverter. This ensured consistent and interference-free power delivery in outdoor conditions, without relying on grid access. The station provided sufficient output for the continuous operation of the full setup.

To minimize the influence of local noise sources, all auxiliary equipment—including the laptop and power station—was positioned behind the microphone line, outside the main acoustic path. The laptop was configured to operate with low fan activity, while the power station was monitored to ensure that measurements were taken only during periods when its internal cooling fan remained inactive. These measures effectively eliminated mechanical and airflow noise contributions from nearby equipment.

For dynamic field measurements, three different unmanned aerial vehicles (UAVs) were employed to represent a representative range of rotorcraft propulsion systems and noise characteristics. The selected drones varied in frame size, rotor configuration, and flight dynamics, as summarized in Table 2.

The tested UAVs represent small multirotor platforms with motor speeds reaching up to approximately 51430 RPM and propeller diameters between 2″ and 8.7″. Based on these parameters, the corresponding blade-passing frequencies range up to about 2.6 kHz. The tested microphones fully cover this frequency range and 96 kHz sampling setup. Although the present work focuses on small consumer-class UAVs, the same measurement methodology can be applied to larger or higher-thrust.

**Figure 2:**
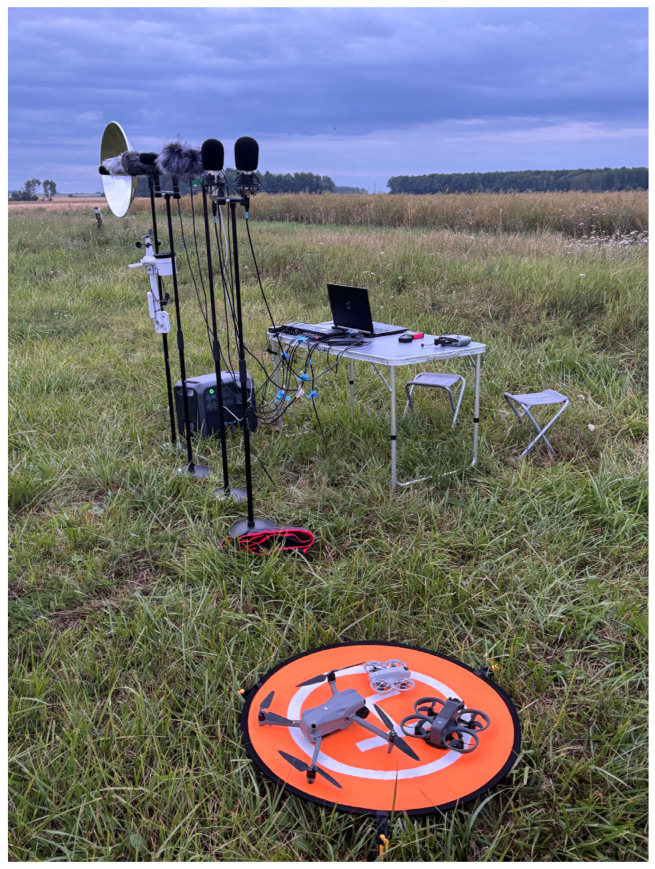
Overview of the field measurement setup.

**Figure 3:**
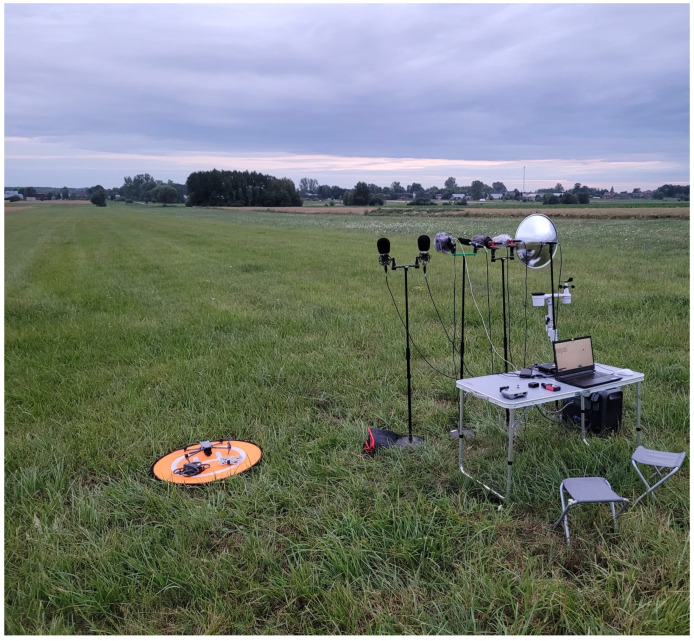
Frontal view of the measurement arrangement.

To complement the technical overview provided above, Figure 2 and Figure 3 illustrate the actual field setup used during the measurement. 

The presented setup was used consistently across all measurement scenarios and test campaigns. Although recordings were conducted on different days and under varying weather conditions, the physical configuration and microphone alignment remained unchanged. To track environmental parameters, a SenseCAP S2120 weather station was deployed during each session to log temperature, humidity, wind speed, and other relevant meteorological data. This ensured high reproducibility and allowed for meaningful comparison of recorded data across time and environmental variations.

### 2.2. Measurement Scenarios

The measurement protocol was designed to capture both spectral characteristics and spatial detection capabilities of the tested microphones in realistic outdoor conditions. Two complementary test scenarios were implemented to achieve this goal.

The first scenario involved recording UAVs during stationary flight (hover) at different fixed distances from the microphone array. This approach enabled the direct analysis of drone-specific acoustic signatures, with a particular focus on identifying dominant frequency components related to rotor design and propulsion dynamics. Recordings were repeated for all UAV models under consistent spatial arrangements to ensure comparability between microphones and between flight sessions. For consistency, all UAV hovers were executed along a single straight baseline radiating from the array origin, at offsets of 1, 2, 5, 10, 15, 20, 30, 40, and 50 m.

While UAVs offered realistic noise profiles, their complex and broadband emissions, combined with highly variable wind conditions, made it difficult to precisely determine each microphone’s detection range based on the signal-to-noise ratio. Wind gusts, in particular, introduced irregular fluctuations in background noise that obscured consistent SNR thresholds. To address this, a second test scenario was introduced, using a narrowband 1 kHz sine wave emitted from a fixed-location speaker. The speaker was calibrated to emit a 1 kHz sine wave at a sound pressure level of 66 dB SPL, measured at a distance of 1 m. This value was selected to approximate the typical broadband acoustic level produced by the DJI Air 3 during hover at the same distance, providing a realistic reference point for evaluating microphone performance. 

The 1 kHz tone was chosen because most microphones are relatively flat and linear in this frequency band, thereby minimizing bias from spectral irregularities. The choice also aligns with common sensitivity and calibrator references, enabling traceable sound-pressure-level calibration. Loudspeakers typically produce low distortion near 1 kHz at moderate levels, and atmospheric absorption around 1 kHz is small and only weakly weather-dependent, thereby improving between-day reproducibility [46]. While wind-induced self-noise is predominantly low-frequency (≲300 Hz), it does not vanish at higher bands [47]: gusts can modulate the 1 kHz tone, generate aeroacoustic “whistle” components into the kilohertz range, and even drive front-end overload that spills energy into the analysis band. Accordingly, probing at 1 kHz reduces—but does not eliminate—wind masking, providing a practical, repeatable test that still differentiates the effectiveness of windshields and other shielding strategies. This reference frequency was not intended to represent the full UAV emission spectrum, but to provide a consistent mid-band stimulus for controlled SNR and range comparison across microphones.

The reference level was first calibrated in a nearly windless, acoustically quiet environment to minimize external influences. This level was then used consistently across all sessions to ensure that the 1 kHz sine wave was emitted at a stable, reproducible amplitude. Distances denote horizontal ground separation measured along a straight, marked baseline whose origin was the geometric center of the microphone stand. Loudspeaker range tests covered 1–250 m along this baseline at the following offsets: 1, 2, 5, 10, 15, 20, 30, 40, 50, 65, 80, 100, 125, 150, 175, 200, 225, and 250 m. The loudspeaker was mounted approximately 1.5 m AGL to match the microphone height, and each position was verified using a mechanical distance wheel. For the UAV trials, the pilot positioned the platform directly above the corresponding ground marker and maintained a stationary hover using GNSS-assisted position hold with barometric altitude hold. Each recording lasted 30 s to stabilize statistics, and the tonal test signal enabled targeted filtering that reduced wind and ambient noise masking.

With both the source and microphones elevated at approximately 1.5 m AGL, propagation is governed by a direct path plus a single ground-reflected path, which can cause distance-dependent level ripples. We did not apply an explicit correction; instead, the source–array geometry was kept fixed for all devices, meaning that the pose and position of the loudspeaker relative to the array were consistent across measurements. Hence, any residual two-path effect is comparable across microphones. Refraction due to temperature and wind gradients was not modeled explicitly; its impact was mitigated by repeating measurements under consistent geometry and reporting meteorological conditions for each session. For the narrow-band range tests, the SNR around 1 kHz was computed against a local noise floor estimated from adjacent bands, with the tone (and visible harmonics) excluded, further reducing sensitivity to ground-reflection artifacts.

All measurements were conducted in open-field environments across five separate test days, selected to capture a range of meteorological conditions—from calm to moderately windy. Although wind speed, gust intensity, wind direction, and ambient temperature varied between sessions, the measurement setup for each propagation direction—i.e., the relative positioning and mounting of microphones—remained consistent to ensure comparability.

The initial test set included five microphone configurations: Behringer B-1, Behringer B-2 PRO, Mozos VMic, RØDE NTG-2 with a standard foam windshield, and the same RØDE NTG-2 mounted within an 18-inch parabolic reflector. All microphones were operated in their intended directional modes, with the Behringer B-2 PRO explicitly set to omnidirectional. Any built-in filters, such as low-cut or high-pass switches, were disabled to preserve the complete broadband response of each device.

The first measurement session, conducted under strong wind conditions and limited to propagation direction C (203° SSW), revealed a significant impact of wind on the performance of specific microphones—particularly the unshielded RØDE NTG-2. In response to these initial findings, two additional configurations were introduced for the remaining sessions: the same NTG-2 microphone was fitted with a RØDE WS6 fur-type windshield, and another was equipped with a RØDE DeadCat windshield. This brought the total number of tested configurations to seven.

The whole measurement campaign was structured to support a stepwise refinement of the test protocol while progressively building toward representative, multi-directional data collection. Following the exploratory first session, two additional sessions were performed using the complete set of seven microphones, again restricted to direction C. These sessions were intentionally conducted under contrasting wind conditions—one low-wind session (mean ≈ 2 m/s, gusts ≈ 4 m/s) and one high-wind session (mean ≈ 5 m/s, gusts ≈ 11 m/s)—to allow a direct assessment of wind shielding effectiveness while keeping the propagation geometry fixed.

The final two sessions constituted the main comparison stage of the campaign. Measurements were conducted under both low and high wind conditions, encompassing all three propagation directions (A, B, and C). This setup enabled a comprehensive evaluation of microphone performance across varied acoustic paths and environmental exposures.

**Figure 4:**
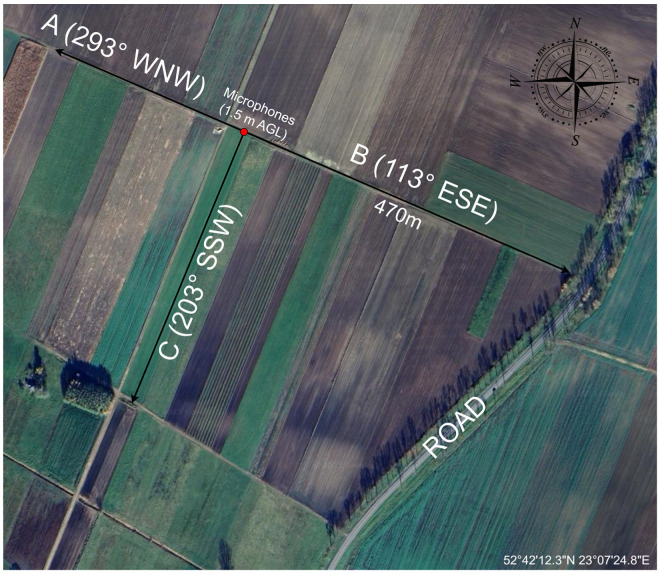
Aerial view of the measurement site with sensor placement and test directions.

The general layout of the test directions and sensor placement is shown in Figure 4. Direction B (113° ESE) was oriented toward a local road, situated several hundred meters beyond the end of the 470 m measurement track. Direction C (203° SSW) extended roughly parallel to that road, while direction A (293° WNW) pointed away from any major infrastructure into quiet agricultural fields with no notable anthropogenic noise sources.

Of the five measurement sessions, only the final four—conducted using the complete set of seven microphones—were included in the subsequent comparative analysis. The initial session, conducted with a limited microphone setup, was excluded due to its exploratory nature and the absence of wind-protected configurations. Environmental conditions recorded during these measurement days are summarized in Table 3. The wind conditions observed during these sessions (mean 1–5 m/s, with gusts up to 11 m/s) correspond to calm-to-moderate outdoor environments typically encountered during UAV operations. This range was considered representative for the intended application, providing realistic yet sufficiently stable conditions for reliable acoustic measurements.

For the spectral analysis of the acoustic signal presented later in this article, data were selected explicitly from recordings made under calm wind conditions. This choice minimized the influence of wind-induced variations in rotor speed, which can occur during active UAV stabilization in gusty environments. All recordings used for the frequency-domain analysis were collected during the fourth measurement session with the full seven-microphone setup.

## 3. Results

This section presents the main findings of the experimental campaign, encompassing both reference signal measurements and acoustic recordings generated by the UAV. The results are divided into two main parts. Section 3.1 focuses on evaluating the effective recording range of each microphone configuration using a standardized 1 kHz sine wave stimulus. Section 3.2 explores the spectral content of UAV emissions and how different microphones respond to their acoustic characteristics under varying environmental conditions. To ensure fair comparison, all recordings were processed with the same offline pipeline across microphones and sessions. The preprocessing and post-processing settings were kept identical for every microphone, with no per-device normalization or tuning. 

### 3.1. Effective Recording Range

As outlined in Section 2.2, the effective recording range of each microphone configuration was assessed using a standardized 1 kHz sine wave emitted from a calibrated loudspeaker at increasing distances. This setup provided a controlled and repeatable baseline for evaluating range detection performance across devices, under realistic outdoor conditions and varying wind intensities. Detection performance was assessed solely as a function of distance (on-axis) using an SNR ≥ 3 dB threshold. No beamforming or off-axis configurations were used.

To ensure meaningful comparison across microphones with different sensitivities and directional properties, all recordings were filtered in the 950–1050 Hz band, corresponding to the 1 kHz calibration tone used in range tests. This narrow band was selected instead of standardized octave or one-third-octave filters to maintain strict spectral focus and reduce overlap with unrelated tonal components or environmental noise, thereby improving repeatability under outdoor conditions. The analysis prioritized relative signal prominence over absolute SPL by computing signal-to-noise ratio values with respect to each microphone’s average background level. The following performance metrics were derived for each configuration:

- Average background noise level, expressed in SPL and computed in the same narrow band as the test tone (950–1050 Hz) from background-only recordings taken at the exact locations and time windows as the signal. Levels are referenced to each microphone’s 1 m recording to remove simple gain differences.
- Mean signal-to-noise ratio, obtained at each distance as the ratio between the band-pass–filtered tone segment and the distance-matched background segment (both 950–1050 Hz), then averaged across all measured distances up to the test limit (or until the detection threshold is crossed).
- Detection range, defined as the distance at which the band-limited SNR first drops below 3 dB; when this occurs between sampled points, the reported distance is linearly interpolated between the two nearest measurements.
- Attenuation slope, the rate at which the band-limited signal level decreases with logarithmic distance (dB/decade), estimated from a straight-line fit over valid points up to the detection limit and referenced to the microphone’s own 1 m level.

Together, these metrics offer a coherent basis for comparing microphone performance across different environmental conditions.

The first scenario focused exclusively on direction C (203° SSW) and included data from two measurement sessions conducted on July 5 and July 12, 2025, under different wind intensities. The first session represented calm conditions, while the second introduced moderate wind exposure. Table 4 presents the extracted performance metrics for each configuration.

The results in Table 4 demonstrate the measurable influence of wind on outdoor acoustic detection, even after targeted bandpass filtering of the 1 kHz test signal. Although the use of narrowband analysis significantly reduced the masking effect of low-frequency wind noise, stronger wind conditions during session two still resulted in a systematic increase of background noise levels—by approximately 5–7 dB—compared to calm conditions.

This increase in ambient noise level resulted in a corresponding decrease in the average signal-to-noise ratio and a reduction in detection range across all microphone configurations. The findings confirm that, despite frequency-selective filtering, wind remains a significant factor in outdoor acoustic measurements, particularly through its impact on turbulence and microphone self-noise.

To assess how performance varies with source direction under similar wind conditions, the following scenario introduces multi-azimuth testing. Table 5 presents results from session 3, conducted under high wind exposure.

Despite a prevailing SSW wind direction, corresponding to direction C, the observed detection ranges were influenced not only by microphone shielding but also by spatial orientation relative to both wind and anthropogenic noise sources. The highest detection distances were generally observed in directions A and C, suggesting that wind gusts did not originate from a constant direction but fluctuated within a southwest sector during the session.

Importantly, direction C was not only aligned with the prevailing wind but also oriented along a road adjacent to the measurement site, which likely facilitated the propagation of environmental noise, such as distant traffic. This effect was especially evident for the parabolic microphone, whose directional gain amplified not only the target sine wave, but also wind-induced noise and roadway sounds arriving from the same axis. As a result, it exhibited notably elevated background SPLs and reduced SNR in this orientation.

In contrast, direction B—perpendicular to the road and oriented more crosswind—showed the lowest detection ranges across most configurations. These observations highlight the compound influence of wind alignment, source directionality, and the propagation of environmental noise. The effectiveness of wind protection is also apparent: microphones equipped with WS6 or DeadCat windscreens consistently maintained higher SNRs and longer detection ranges compared to their unshielded counterparts. Additionally, the parabolic dish—despite its directional sensitivity—offered some passive shielding from lateral wind exposure, contributing to improved performance in directions less aligned with the wind.

To isolate the effect of spatial orientation under minimal wind interference, a final measurement session was conducted under calm weather conditions (session 4). This scenario enabled the evaluation of directional sensitivity and ambient noise exposure across all configurations, eliminating the confounding influence of strong wind. The results for all three directions are summarized in Table 6.

Under low wind conditions (mean 1 m/s), the differences in detection performance were primarily influenced by microphone orientation and environmental background sources, rather than by wind-induced turbulence. Direction A consistently yielded the lowest background noise level and highest SNR values, likely due to minimal external interference and favorable propagation conditions. In contrast, direction B—pointing toward nearby road infrastructure—showed the highest background noise levels and the shortest detection ranges across most configurations. Direction C, despite elevated ambient noise levels, maintained comparable detection ranges to A, suggesting efficient signal propagation along the road axis. These results confirm that even under calm conditions, spatial orientation and landscape features can significantly modulate effective microphone range.

The results across all test scenarios reveal several consistent trends in microphone performance under varying wind conditions and spatial orientations. Most notably, detection ranges were substantially more extended in the direction opposite to the road. In contrast, measurements along or toward the road exhibited pronounced reductions in both SNR and effective range. This underscores the compound effect of environmental noise propagation and source alignment.

Wind presence emerged as a dominant limiting factor. Even moderate gusts resulted in elevated background noise and reduced detection distances across all devices. Wind protection solutions—such as the WS6 and DeadCat windshields—did not notably affect signal attenuation under calm conditions but proved highly beneficial under wind exposure, improving both SNR and detection range. 

To provide a global view of performance consistency across all test conditions, Table 7 summarizes the inter-session mean ± standard deviation of key acoustic metrics for each microphone configuration. These aggregated values reflect combined variability due to wind, direction, and session-specific factors.

The aggregated results indicate that directional microphones equipped with windshields (NTG-2 + WS6 or DeadCat) and the parabolic NTG-2 consistently achieved the highest mean SNRs (≈10–11 dB) and the longest detection ranges (≈85–95 m), confirming their superior resilience to environmental variability. In contrast, the omnidirectional Behringer B-2 and the compact Mozos VMic exhibited both lower average SNRs and larger inter-session deviations, reflecting higher sensitivity to wind and ambient noise fluctuations. Overall, the inter-session standard deviations (typically 2–3 dB for SNR and 40–45 m for range) quantify the natural variability observed across outdoor sessions under changing wind and background noise conditions, thereby strengthening the statistical validity of the comparison.

**Figure 5:**
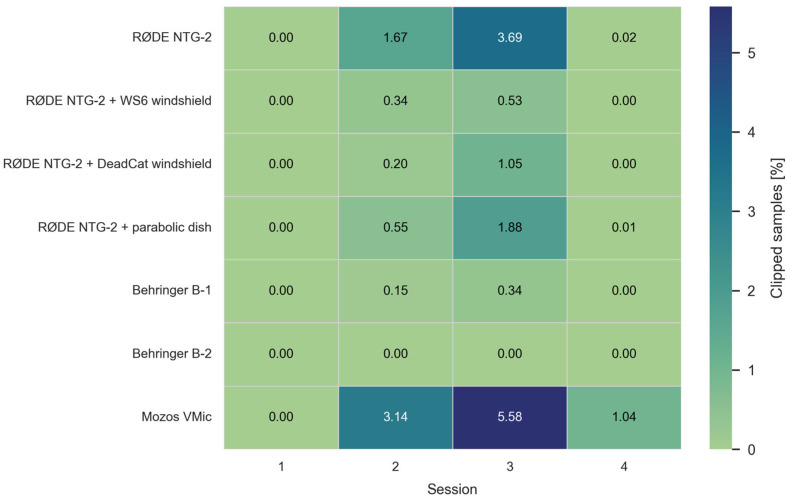
Clipping incidence by microphone and session.

To verify that range and SNR estimates were not biased by saturation, we audited every recording for clipping, treating any sample with normalized magnitude ∣x∣ ≥ 0.999 (≈−0.01 dBFS) as effectively full scale. The 0.999 guard band—slightly below unity—captures hard-limited peaks that can appear just under 1.0 after PCM-to-float conversion or rounding, avoiding false negatives. We aggregated the rates per microphone and session (Figure 5). Clipping emerged primarily as a wind-driven transient, being negligible in calm sessions (1 and 4). It concentrated on the high-wind, multi-azimuth session 3. Headroom tracked shielding and front-end design—windshielded cardioids (NTG-2 + WS6/DeadCat) stayed at or below ~1% across sessions; the parabolic NTG-2 showed occasional moderate overload in gusts; bare NTG-2 and Mozos VMic were most susceptible; large-diaphragm Behringers (B-1/B-2) were near zero. Overall rates were small relative to total sample counts, indicating that the reported detection ranges and SNRs are not artifacts of clipping. 

**Figure 6:**
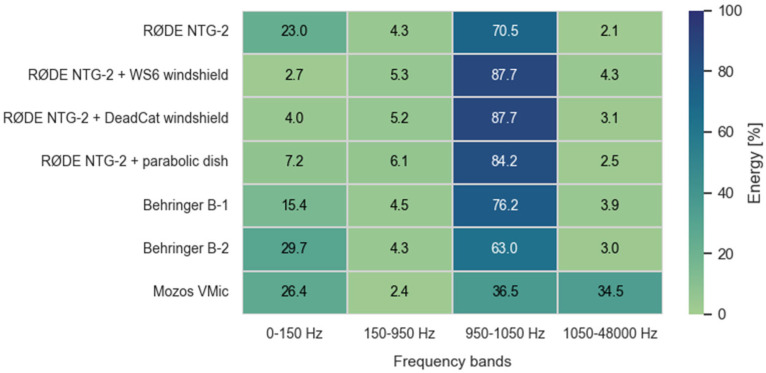
Energy distribution under low wind-session 1.

**Figure 7:**
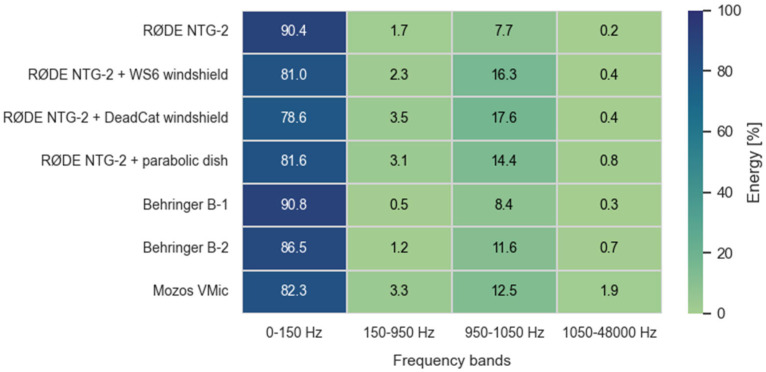
Energy distribution under high wind-session 2.

The spectral distribution of energy across key frequency bands offers further insight into how microphones respond to wind-induced noise and signal content. Energy in each frequency band was obtained by summing the squared FFT magnitudes across all bins within the band and expressing the result as a percentage of the total broadband energy (0–48 kHz). The unequal bandwidths were selected to isolate wind-dominated (<150 Hz), transitional (150–950 Hz), signal (950–1050 Hz), and high-frequency (>1050 Hz) regions. Figure 6 and Figure 7 illustrate the average energy distribution across these frequency regions under low and high-wind conditions, respectively.

Under calm conditions, most energy is concentrated in the 950–1050 Hz band, corresponding to the test signal, with minimal low-frequency interference. However, under high wind exposure, unshielded microphones (e.g., bare NTG-2) exhibit strong energy components below 150 Hz—characteristic of wind-induced turbulence.

In contrast, configurations using WS6 or DeadCat windscreens show marked suppression of low-frequency energy while preserving the main tone’s prominence. The parabolic dish similarly improves signal focus, although its broadband gain also slightly increases the capture of higher-frequency energy. These spectral effects are consistent with its overall performance: the parabolic dish configuration consistently delivered the highest or near-highest detection ranges across all test scenarios, typically outperforming standard directional microphones. While the reflector amplified both the target signal and ambient noise—especially in the presence of wind or distant background sources—it nevertheless resulted in systematically elevated SNR values. This indicates that its directional gain effectively favored on-axis sound capture, enhancing signal clarity even when absolute range improvements were modest. Among the tested devices without advanced wind protection, the Behringer B-1 exhibited notably resilient performance. Despite being equipped only with a basic foam windscreen, it maintained relatively high SNRs and detection ranges-potentially due to its capsule geometry or internal acoustic damping, which limited wind-induced interference. In contrast, the Behringer B-2, which features an omnidirectional pickup pattern, consistently demonstrated reduced detection capability and lower SNR values across all scenarios.

The Mozos VMic exhibited the highest susceptibility to ambient and wind-related noise, despite being fitted with both foam and fur windshields. One practical limitation stems from its two-channel output, which reduces the number of microphones that can be simultaneously connected to a multi-channel audio interface—thereby constraining experimental setups. Additionally, the presence of a manual gain control introduces variability in output level, complicating reproducible calibration. Although the gain was set to maximum for range testing, the microphone’s overall performance remained limited, likely due to internal design factors such as preamplifier quality or capsule sensitivity, rather than shielding alone.

Attenuation slopes provided additional insight into how different configurations affected the propagation and perceived loss of signal strength over distance. Steeper slopes (up to −20 dB/decade) were typically observed under calm conditions and in directions free from primary noise sources, reflecting a more idealized, free-field decay of sound pressure. Conversely, shallower slopes (e.g., −13 dB/decade or less) emerged more frequently in windy conditions or when microphones faced dominant background sources, where elevated noise masked the decay curve and reduced the effective contrast between the signal and background. Notably, the parabolic reflector consistently exhibited the flattest slopes across scenarios—indicating that its directional gain not only amplified the target signal but also helped preserve its level across longer distances.

### 3.2. Spectral Analysis of UAV Acoustic Signatures

Beyond range metrics, this section examines how each microphone captures UAV-specific tonal content relative to its own background noise. In particular, microphones with a lower low-frequency noise floor (0–300 Hz) and stronger mid-band tonal excess (i.e., energy integrated over a harmonic comb anchored at blade passage frequency) tend to sustain higher SNR with distance. Accordingly, the results in this section should broadly align with the detection-range ordering reported in Section 3.1, except under wind-dominated conditions or unfavorable source–array orientations. These trends are closely linked to the spectral structure of UAV noise. Small multirotor UAVs emit complex acoustic signatures composed of both tonal components and broadband noise. These sounds are primarily generated by the rotation of propellers and brushless electric motors, and their characteristics vary depending on drone size, structural geometry, and flight dynamics. Even during steady hovering, UAVs produce non-stationary emissions due to continuous feedback-driven adjustments in motor RPM to maintain stability.

The acoustic characteristics of such drones are shaped not only by their operational parameters but also by their physical configuration. Quadcopter designs typically feature four identical propulsion units (motors and propellers) mounted symmetrically, which results in predictable harmonic structures. These include strong periodic components related to the blade passage frequency, as well as higher-order harmonics from each motor–propeller unit. The BPF corresponds to the rate at which propeller blades pass a fixed point in space and can be expressed by the following equation:

(1) $$f_{0}=\;\frac{N\times RPM}{60}$$

where *N* is the number of blades per propeller and *RPM* is the rotation speed in revolutions per minute. This frequency typically dominates the tonal content of the UAV’s acoustic signature and determines the spacing between successive harmonics. Due to the rigid and symmetrical layout, these harmonics are often more narrowly distributed and stable compared to those found in organic sound sources, such as human speech.

Additionally, structural vibrations, airflow interactions, and subtle differences in motor loading introduce further modulation components, which give rise to subharmonics and spectral variations. While drone maneuvers or accelerations can cause momentary shifts in the spectral content, even in static flight, the combination of mechanical precision and aerodynamic effects results in a rich yet structured acoustic profile characteristic of multirotor UAVs.

**Figure 8:**
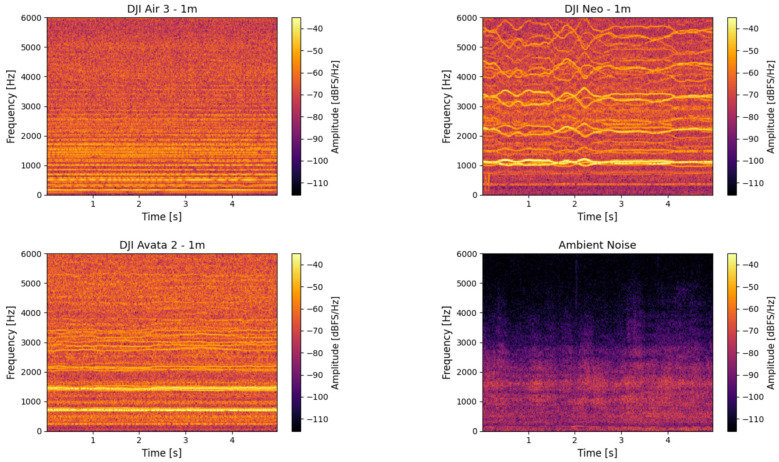
Spectrograms of UAV acoustic emissions during hovering flight for DJI Air 3, DJI Avata 2, DJI Neo, and ambient background noise (silence).

To illustrate these spectral characteristics in practice, Figure 8 presents spectrograms of three UAV models-DJI Air 3, DJI Neo, and DJI Avata 2-recorded during hovering at a distance of 1 meter using the RØDE NTG-2. While the figure shows results obtained with this microphone, identical tonal components and frequency structures were consistently observed across all other tested microphones. The spectrograms varied in terms of signal strength and wind-related noise, depending on the microphone type and shielding, but the underlying frequency content remained unchanged. The RØDE NTG-2 results were selected for illustration due to their clarity and representativeness.

These examples illustrate the broadband nature of UAV emissions, the presence of tonal bands associated with motor and propeller harmonics, and the variations in spectral energy concentration among different types of drones. All spectrograms are expressed in decibels relative to full scale per hertz (dBFS/Hz), where 0 dBFS corresponds to the maximum representable digital amplitude of the recorder.

The spectrograms in Figure 8 reveal that all three UAVs generate broadband acoustic emissions, with energy concentrated predominantly in the lower frequency range, below 2 kHz. However, apparent differences can be observed across models. The DJI Air 3 exhibits stable tonal bands with harmonics spaced at regular intervals, suggesting consistent motor speed and minimal vibrational interference. The DJI Neo, in contrast, presents a more fragmented spectral pattern, characterized by irregular harmonic spacing and increased high-frequency content, which may indicate unstable hovering or fluctuating rotor speeds. Notably, the harmonic components for this model, although lower in energy, extend up to approximately 15 kHz, indicating a broader spectral footprint compared to the other UAVs. Meanwhile, DJI Avata 2 concentrates most of its acoustic energy in the lowest frequency bands and shows fewer distinct harmonic structures.

To quantify UAV-specific content, we estimated one-sided power spectral densities (PSDs) using the Welch method from 30 s hover segments for each microphone and from an immediately adjacent background noise segment recorded with the same geometry (Hann window; segment length, N = 32,768 samples; 50% overlap). This parameterization was chosen after exploratory testing of alternative settings (4096–65,384 samples, 25–75% overlap) and provides an optimal trade-off between frequency resolution (~2.93 Hz) and temporal stability under variable wind conditions. Shorter windows degraded harmonic separation and increased PSD variance, while longer ones improved resolution at the expense of robustness to transient background fluctuations. The selected configuration ensured that UAV-specific tonal peaks remained well resolved while minimizing sensitivity to non-stationary ambient noise. The following metric was computed separately for each microphone:

(2) $$∆{PSD}_{m}\left(f\right)\;=\;10{log}_{10}\left(\frac{{PSD}_{UAV}(f)}{{PSD}_{bg}(f)}\right)[dB]$$

where *PSD_UAV_(f)* and *PSD_bg_(f)* denote the Welch PSDs of the UAV and background noise segments, respectively, values > 0 dB indicate excess acoustic energy attributable to the UAV. Candidate tonal components were then located on *ΔPSD_m_(f)* after light Gaussian smoothing (σ ≈ 20 Hz). Peaks were required to have a minimum prominence of 6 dB. For each detected peak, the tonal band was defined by the two -6 dB crossings of *ΔPSD_m_(f)* around the peak.

**Figure 9:**
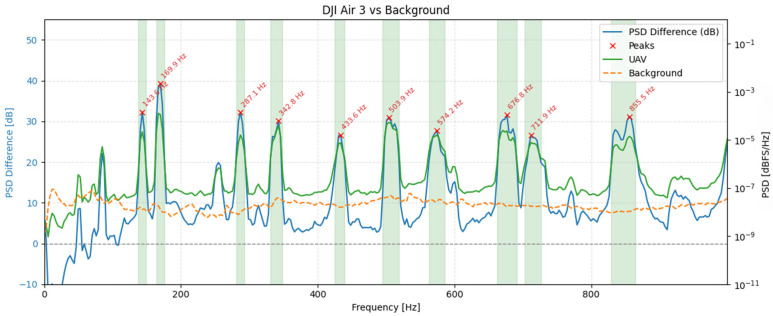
PSD comparison between the DJI Air 3 during hovering and ambient background noise.

**Figure 10:**
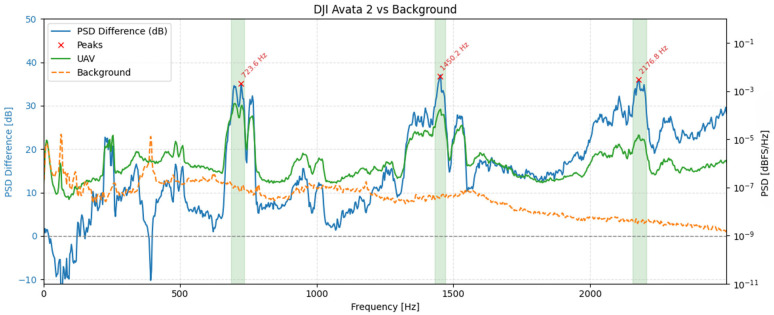
PSD comparison between the DJI Avata 2 during hovering and ambient background noise.

**Figure 11:**
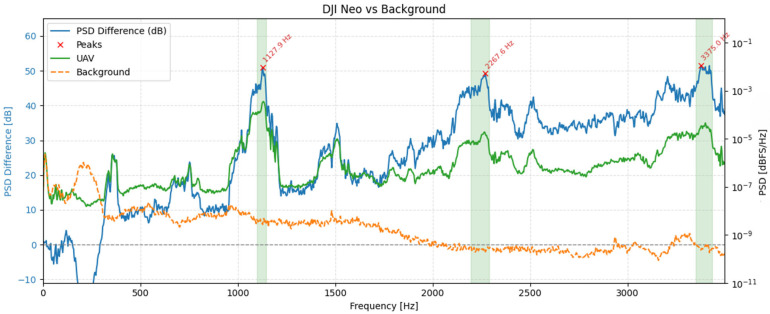
PSD comparison between the DJI Neo during hovering and the ambient background noise.

Figure 9, Figure 10 and Figure 11 show, for each platform, the UAV (green) and background (orange) PSDs together with the differential spectrum *ΔPSD_m_(f)* (blue). Red markers denote peaks selected by the ≥ 6 dB prominence criterion, and shaded regions indicate the automatically derived ±6 dB tonal bands. These plots highlight frequency ranges where UAV energy rises above the background noise.

The spectral profiles of the three UAV platforms reveal distinct yet structurally consistent acoustic patterns. For the DJI Air 3, two separate families of harmonic peaks can be observed: one aligned around ~170 Hz and its integer multiples, and another originating near ~144 Hz. This dual-harmonic structure likely stems from slight speed differences between rotor pairs, suggesting desynchronization or intentionally varied RPMs for control stability. In contrast, the DJI Avata 2 exhibits fewer discrete peaks but broader spectral regions centered near ~724 Hz, 1450 Hz, and 2177 Hz. These wider bands may reflect overlapping contributions from multiple rotors operating at close but non-identical frequencies, or the influence of modulated motor loads and frame dynamics. DJI Neo presents the most broadband character, with dominant wide peaks at ~1128 Hz, 2268 Hz, and 3375 Hz. The absence of narrow, harmonically spaced tones suggests greater spectral overlap or frequency modulation across rotors. Despite these differences, all UAVs exhibit tonal components with clearly elevated power above background noise levels, confirming the presence of UAV-specific acoustic signatures. 

While Figure 9, Figure 10 and Figure 11 visually highlight the locations of UAV tonal components, they also represent quantitative results, as numerical spectral metrics were computed for each case. For every UAV-microphone pair and for each recorded distance, we compare the UAV segment with a time-adjacent background segment of identical geometry and compute three complementary metrics:

- Harmonic SNR [dB]–the ratio of UAV to background spectral power integrated only within narrow bands centered at integer multiples of the blade-pass frequency $f_{0}$. The set of bands is anchored at $f_{0}$ estimated from the 1 m recording (through cepstral analysis) and spaced at its harmonics; each band spans ±8% of its center frequency to tolerate small RPM fluctuations. It is calculated by the following expression:
  (3) $${SNR}_{harm}={10log}_{10}\left(\frac{\int_{B_{H}}{PSD}_{UAV}\left(f\right)df}{\int_{B_{H}}{PSD}_{BG}\left(f\right)df}\right)[dB]$$
  where *PSD_UAV_(f)* and *PSD_BG_(f)* are the one-sided Welch *PSDs* and *B_H_* denotes the union of all harmonic windows:
  (4) $$B_{n}=[(1-p)nf_{0},(1+p)nf_{0}]$$
  with *p* = 0.08. This metric isolates UAV-specific tonal energy while de-emphasizing broadband clutter.
- Wideband SNR [dB]–computed analogously, but integrated over the full analysis range [0, *F*_max]_ with *F*_max_ = 48 kHz, reflecting overall prominence of UAV sound without harmonic priors.
- LF floor [dB]–the median background noise level in the low-frequency (LF) band (0–300 Hz), representative of wind/handling/flow noise.

For each UAV-microphone pair, the metrics were averaged across all available distances (1–50 m) to avoid undue influence of any single range point. Results are reported separately for each UAV to preserve platform-specific spectral characteristics.

On DJI Air 3 (Table 8), harmonic SNRs cluster tightly around modest values across all NTG-2 configurations, indicating relatively weak tonal structure for this platform. Adding WS6 or DeadCat primarily improves wideband SNR by a few decibels and lowers the LF floor by a similar margin, consistent with better suppression of wind-borne noise. The parabolic dish delivers a low LF floor comparable to the large-diaphragm Behringer microphones, while the Mozos VMic exhibits the highest LF floor and the lowest wideband SNR.

For DJI Avata 2 (Table 9), harmonic energy is markedly stronger: harmonic SNR rises into the mid-teens and above for most microphones. Windshields again boost wideband SNR relative to the bare NTG-2 without materially changing the harmonic SNR ordering. Large-diaphragm condensers achieve the quietest LF floors, reflecting good immunity to low-frequency contamination during hover.

DJI Neo (Table 10) shows the most pronounced tonal signature. The harmonic SNR is highest for the WS6 and parabolic configurations, with the bare NTG-2 trailing slightly; the wideband SNR follows the same pattern. LF floors lie in the −70 to −78 dB range, with Behringer microphones again among the quietest at low frequencies.

Across platforms, three patterns emerge. First, harmonic SNR—the most relevant figure for tonal detectors—shows only minor differences between NTG-2 variants on Air 3 and Avata 2, but clear gains for WS6/parabola on Neo. Second, wideband SNR benefits consistently from WS6 (and, to a lesser extent, DeadCat), indicating that overall signal prominence in mixed noise is often limited by the LF background noise rather than by the capsule’s ability to capture tones. Third, microphones with inherently lower LF floors (parabolic and large-diaphragm condensers) are advantaged whenever wind/flow noise dominates.

## 4. Microphone Array Development

The findings presented in this study provide a strong experimental basis for designing an acoustic UAV-detection microphone array. While range testing differentiated microphones in terms of signal strength, attenuation, and wind resilience, the spectral analysis of UAV emissions identified the frequency regions where sensitivity is most valuable. All tested microphones provided adequate response in those bands.

Quantitatively, the RØDE NTG-2 with WS6 windshield offered the most reliable performance across wind and orientation while maintaining competitive tonal sensitivity:

- Under high wind (session 2), WS6 extended the detection range over bare NTG-2 from 34.8 m to 45.6 m (+31%; Table 4). In multi-azimuth high wind (session 3), it increased range from 72.0 m to 96.6 m in direction A (+34%) and from 28.3 m to 65.4 m in direction B (+131%), with near parity in C (Table 5). Under calm conditions (session 4), ranges were broadly comparable, with small WS6 gains in A/C and a slight deficit in B (Table 6).
- In hover across all three UAVs, WS6 consistently lowered the LF floor relative to bare NTG-2 (Air 3: −78.3 vs. −75.5 dB; Avata 2: −77.3 vs. −75.8 dB; Neo: −75.2 vs. −72.1 dB) and increased or matched wideband SNR (+1.8, +4.4, +2.6 dB; Table 8, Table 9 and Table 10).
- Compared with DeadCat, WS6 matched or exceeded detection range under wind (e.g., session 2: 45.6 vs. 35.7 m; session 3, dir. B: 65.4 vs. 37.1 m) while maintaining equal-or-higher wideband SNR and a consistently lower LF floor across UAVs; DeadCat could at times suppress <150 Hz energy slightly more in the strongest gusts, but its LF floor and range were less robust overall (Table 4, Table 5, Table 6, Table 7, Table 8, Table 9 and Table 10).

The parabolic NTG-2 configuration achieved a very low LF floor and strong on-axis tonal reach. Large-diaphragm Behringer microphones were LF-quiet, but in our tests, they delivered shorter ranges than the NTG-2 variants and are less practical for a compact array. The Mozos VMic exhibited elevated self and ambient noise with a limited range. For the array core, we therefore selected NTG-2 + WS6. Although pairing WS6 with a parabolic reflector can appear top-performing for a single channel, we exclude reflectors from the baseline array for three reasons relevant to detection and future localization:

- A narrow, frequency-dependent beam improves on-axis range but sacrifices hemispherical coverage and makes first-hit detection sensitive to pointing accuracy.
- The reflector path introduces angle and frequency-dependent phase and path-length offsets and greater unit-to-unit variability. This degrades the cross-channel phase coherence required by phase-sensitive array processing (e.g., time difference of arrival (TDOA) estimation and steered response power with phase transform (SRP-PHAT) beamforming) unless each element is individually calibrated and adjusted.
- Despite low steady-state LF levels, the lightly shielded focal capsule remains vulnerable to gust-induced transients (clipping/pumping) and added windage observed in our range tests, complicating unattended field operation.

Guided by these findings, we designed an eight-channel array in a vertically stacked dual-tier layout to maximize hemispherical coverage and directional diversity. The use of eight microphones represented a practical and acoustic compromise between coverage, directivity, and hardware complexity. This configuration ensures full 360° azimuthal sampling with four microphones per tier, while maintaining compatibility with a standard 8-channel audio interface used in field recording. Increasing the number of elements would only marginally improve spatial resolution at the cost of higher system complexity and reduced portability.

The lower tier, with microphones spaced at 90° intervals and tilted 10° upward, is optimized for detecting horizontally distant UAVs. The upward angle compensates for the low mounting height of the array, ensuring sensitivity to sound sources slightly above the horizontal plane. The upper tier, rotated 45° in azimuth and tilted at 45°, extends coverage into higher elevation angles, supporting detection of UAVs flying overhead or at steeper approach angles. Together, the two tiers form a compact structure that approximates hemispherical acoustic coverage around the array center.

This geometric arrangement provides near-hemispherical acoustic coverage around the array center, effectively capturing sound sources arriving from a wide range of directions. By combining microphones tilted at shallow (10°) and steep (45°) upward angles across two vertically separated tiers, the system maintains sensitivity to both low-elevation and high-elevation signals.

The array geometry was derived from the directional response characteristics of the NTG-2 microphones and analytical coverage considerations, ensuring complementary overlap between individual cardioid lobes. Preliminary field validation with UAV flyovers confirmed uniform sensitivity and stable SNR balance across vertical elevation angles. This layout minimizes directional blind spots—particularly those at overhead or oblique angles—and ensures consistent directional selectivity due to the inherent polar patterns of the NTG-2 microphones. The result is a spatially balanced capture field that maximizes the probability of UAV detection regardless of flight path orientation, while preserving the angular resolution necessary for future localization or tracking tasks.

**Figure 12:**
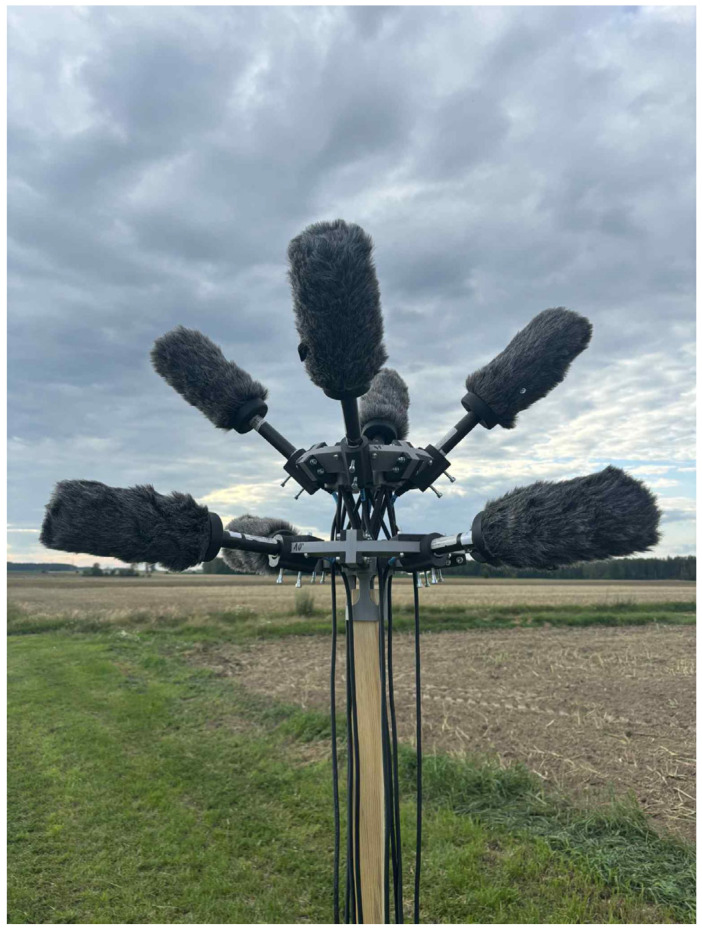
Dual-layer NTG-2 microphone array with windshields.

**Figure 13:**
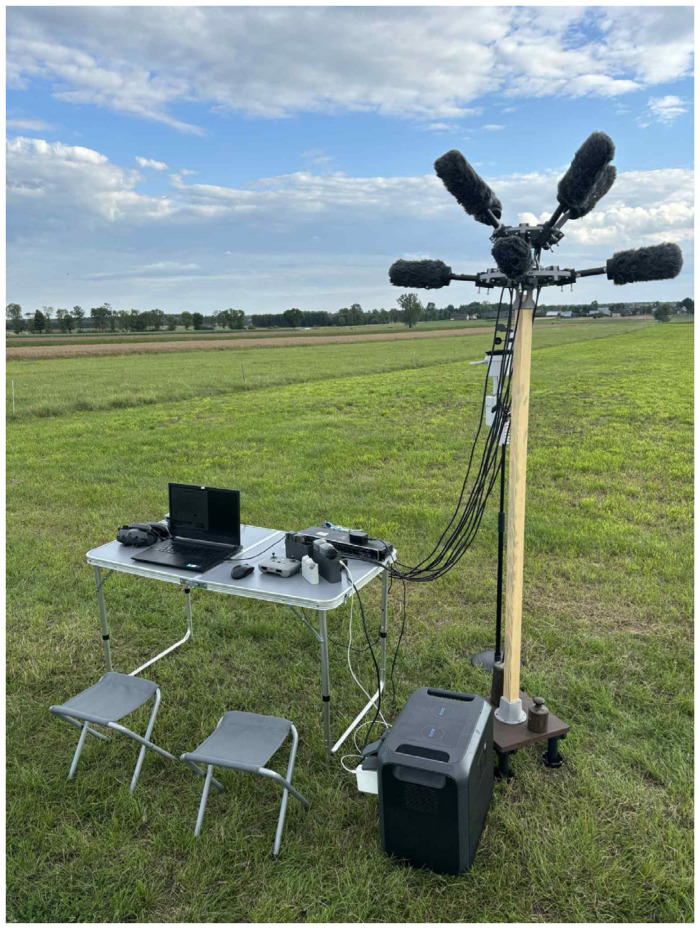
Field deployment of the array with a full recording setup.

Figure 12 and Figure 13 show the completed microphone array during initial field deployment tests. The structure was fabricated using 3D printing techniques, enabling lightweight construction and precise alignment of all eight microphones. The dual-tier frame is mounted on a wooden mast for stable elevation, and the entire setup is designed for rapid assembly and vertical adjustment using foldable, terrain-adaptable legs.

Beyond its geometric and acoustic design, the array was developed as a modular, field-deployable research platform for synchronized, multi-channel recording of UAV emissions in realistic outdoor environments. Its construction enables rapid deployment across varied terrains and supports data acquisition across diverse flight profiles, distances, and noise conditions.

The collected signals aim to capture both the spatial and spectral characteristics of UAV acoustics, forming the foundation for future datasets used in detection, classification, and localization tasks, including those that leverage AI-based methods.

Looking ahead, we will evaluate adding selected parabolic units as auxiliary long-range directional modules where extreme range is required. We are also considering telescopic mounts (e.g., RØDE Boompole-style) to allow adjustable vertical/radial positioning, further adapting the array to diverse deployment scenarios.

## 5. Conclusions

This study presented a structured, detection-centric evaluation of microphone performance for outdoor UAV acoustics, combining calibrated range tests with spectral analysis of real UAV emissions across three platforms. Using a common set of hardware metrics—signal-to-noise ratio, effective detection range, distance-attenuation slope, and the low-frequency background noise floor—we characterized how capsule directivity and wind protection translate into practical detectability (Table 4, Table 5, Table 6, Table 7, Table 8, Table 9 and Table 10).

All microphones captured the relevant UAV spectrum, yet differed markedly in range and wind susceptibility. Across wind and orientation, the RØDE NTG-2 with WS6 windshield provided the most consistent trade-off: it extended detection range relative to the bare NTG-2 under wind, reduced the LF floor, and matched or exceeded wideband SNR on all three UAVs. Parabolic focusing yielded very low LF floors and strong on-axis tonal reach, but narrowed angular coverage and showed vulnerability to gust-induced transients. Large-diaphragm Behringer models were LF-quiet but did not capitalize on that advantage to achieve longer ranges. At the same time, the Mozos VMic exhibited the shortest ranges.

Environmental factors were decisive. Wind elevated the background noise and steepened performance differences; source–array orientation modulated outcomes, with directions away from road noise yielding higher SNR and longer ranges than road-facing measurements. These observations align the range results with the hover spectra: microphones that lowered the LF floor and preserved mid-band tonal energy maintained usable SNR over distance.

Guided by these findings, we implemented a modular, dual-tier, eight-channel array of NTG-2 microphones with WS6 windshields, providing near-hemispherical coverage and consistent directional sensitivity for synchronized, multi-channel field recording. The resulting platform establishes a reproducible hardware baseline and a metric framework for future UAV-acoustic datasets and algorithms.

The conducted tests provide a practical and reproducible assessment of microphone performance in real outdoor conditions; however, several limitations should be noted. Measurements were carried out only under calm and high-wind regimes, without intermediate meteorological states that could reveal transitional effects. All tests were performed in open-field environments to reflect realistic UAV-detection scenarios, which naturally limited acoustic isolation and control over ambient noise. Minor background artifacts—such as low-level wind turbulence or device noise—could not be entirely eliminated, although their impact on SNR estimation was negligible.

The evaluation also covered a finite set of microphone configurations. Future studies may include additional variants, such as parabolic dishes with integrated windshields or alternative capsule geometries, to examine how design trade-offs influence detection range and low-frequency noise resilience.

## Figures and Tables

**Table 1:** Measurement hardware used in this study.

Device	Model	Key Specifications	Notes/Use
Shotgun mic	RØDE NTG-2 [41]	Supercardioid; 20–20 kHz; sensitivity ≈ −36 dBV/Pa; self-noise ≈ 18 dBA	Tested with foam, RØDE WS6, and DeadCat; also with 18″ parabolic dish
Shotgun mic	Mozos VMic	Cardioid; 50–20 kHz; sensitivity ≈ −36 dBV/Pa; self-noise ≈ 14 dBA	With a furry windshield
Condenser mic	Behringer B-1 [42]	Large-diaphragm; Cardioid; 20–20 kHz; sensitivity ≈ −34 dBV/Pa; self-noise ≈ 13 dBA	—
Condenser mic	Behringer B-2 PRO [43]	Dual-diaphragm; 20–20 kHz; sensitivity ≈ −37 dBV/Pa; self-noise ≈ 18 dBA	Switchable polar patterns

**Table 2:** UAV platform characteristics relevant to acoustic analysis.

Model	Rotor Configuration	Propeller Diameter	Max RPM	Mass (g)
DJI Air 3	4 rotors, 2-blade propellers	221 mm (~8.7″)	8400	720
DJI Avata 2	4 rotors, 3-blade propellers	76.2 mm (~3″)	51430	377
DJI Neo	4 rotors, 3-blade propellers	50.8 mm (~2″)	36570	135

**Table 3:** Meteorological conditions during measurements.

Measurement Day	Temperature [°C]	Wind Speed [m/s]		Wind Direction
		Mean	Gusts	
05.07.2025 (session 1)	25	2	4	SSW
12.07.2025 (session 2)	21	5	11	SSE
24.07.2025 (session 3)	24	5	11	SSW
26.07.2025 (session 4)	24	1	3	NNW

**Table 4:** Microphone comparison under low and high wind conditions (sessions 1 and 2).

Microphone	Wind Level	Avg. Noise [dB]	Avg. SNR [dB]	Detection Range [m]	Attenuation Slope [dB/decade]
RØDE NTG-2	Low	32.3	9.4	83.9	−14.4
	High	37.2	6.4	34.8	−16.2
RØDE NTG-2 + WS6 windshield	Low	32.3	9.8	78.8	−15.5
	High	37.5	7.2	45.6	−14.1
RØDE NTG-2 + DeadCat windshield	Low	31.8	9.8	79.3	−15.8
	High	38.2	6.7	35.7	−15.8
RØDE NTG-2 + parabolic dish	Low	36.9	10.0	93.5	−11.7
	High	42.4	7.9	42.1	−12.9
Behringer B-1	Low	32.4	9.1	75.4	−14.0
	High	39.4	7.5	43.2	−14.9
Behringer B-2	Low	34.9	8.4	69.0	−13.3
	High	40.0	6.4	38.3	−14.3
Mozos VMic	Low	34.1	8.2	78.1	−13.1
	High	39.2	6.3	36.7	−15.0

**Table 5:** Microphone comparison under high wind conditions (session 3).

Microphone	Direction	Avg. Noise [dB]	Avg. SNR [dB]	Detection Range [m]	Attenuation Slope [dB/decade]
RØDE NTG-2	A	33.8	8.7	72.0	−16.1
	B	37.3	6.1	28.3	−17.0
	C	36.1	7.4	47.4	−14.5
RØDE NTG-2 + WS6 windshield	A	26.3	12.0	96.6	−16.3
	B	32.5	9.2	65.4	−18.5
	C	35.6	7.7	48.5	−14.8
RØDE NTG-2 + DeadCat windshield	A	27.5	11.8	93.3	−16.1
	B	32.8	8.3	37.1	−18.5
	C	34.7	8.0	48.4	−14.8
RØDE NTG-2 + parabolic dish	A	30.3	12.5	98.2	−14.4
	B	32.4	9.5	59.7	−17.5
	C	40.3	8.0	69.0	−11.3
Behringer B-1	A	29.2	12.7	98.6	−17.4
	B	38.1	7.2	33.6	−16.4
	C	37.3	7.5	45.3	−13.1
Behringer B-2	A	37.7	7.9	37.1	−17.4
	B	44.2	5.4	27.6	−14.6
	C	36.6	7.3	44.8	−13.6
Mozos VMic	A	45.5	6.1	38.7	−17.8
	B	47.6	4.6	21.7	−14.0
	C	45.9	7.0	33.2	−15.1

**Table 6:** Microphone comparison under low wind conditions (session 4).

Microphone	Direction	Avg. Noise [dB]	Avg. SNR [dB]	Detection Range [m]	Attenuation Slope [dB/decade]
RØDE NTG-2	A	18.1	17.1	133.8	−20.5
	B	29.7	10.7	72.8	−17.0
	C	29.9	12.1	103.9	−13.4
RØDE NTG-2 + WS6 windshield	A	17.4	17.4	138.7	−20.3
	B	31.5	10.1	68.3	−17.3
	C	31.6	12.7	108.1	−13.6
RØDE NTG-2 + DeadCat windshield	A	20.8	16.0	130.8	−19.4
	B	28.4	10.7	71.3	−18.1
	C	29.6	13.0	106.0	−13.5
RØDE NTG-2 + parabolic dish	A	21.0	16.4	136.6	−18.3
	B	39.0	10.0	74.6	−13.6
	C	31.2	13.3	108.9	−13.8
Behringer B-1	A	27.9	14.1	117.1	−17.6
	B	37.2	9.6	68.1	−15.3
	C	35.4	11.4	72.6	−13.7
Behringer B-2	A	26.8	12.8	100.3	−19.3
	B	35.3	8.9	65.2	−16.4
	C	37.9	7.2	46.9	−12.8
Mozos VMic	A	25.2	12.4	105.1	−19.2
	B	32.7	8.1	46.6	−18.2
	C	35.8	11.2	77.2	−12.5

**Table 7:** Inter-session mean ± standard deviation metrics.

Microphone	Noise [dB]	SNR [dB]	Detection Range [m]	Attenuation Slope [dB/decade]
RØDE NTG-2	31.9 ± 6.3	9.8 ± 3.5	80.1 ± 44.4	−15.8 ± 2.4
RØDE NTG-2 + WS6 windshield	30.6 ± 6.2	10.7 ± 3.4	86.1 ± 42.9	−16.6 ± 2.2
RØDE NTG-2 + DeadCat windshield	30.5 ± 5.2	10.5 ± 3.0	81.9 ± 44.6	−16.5 ± 2.0
RØDE NTG-2 + parabolic dish	34.2 ± 6.9	11.0 ± 2.9	91.7 ± 39.6	−14.2 ± 2.5
Behringer B-1	34.6 ± 4.3	9.9 ± 2.6	79.9 ± 42.6	−15.2 ± 1.8
Behringer B-2	36.7 ± 5.0	8.0 ± 2.2	53.7 ± 23.5	−15.2 ± 2.3
Mozos VMic	42.9 ± 14.9	5.9 ± 4.9	65.6 ± 44.5	−12.0 ± 7.7

**Table 8:** DJI Air 3—microphone performance during hover (mean over 1–50 m).

Microphone	Harmonic SNR [dB]	Wideband SNR [dB]	LF [dB]
RØDE NTG-2	5.4	3.7	−75.5
RØDE NTG-2 + WS6 windshield	5.7	5.6	−78.3
RØDE NTG-2 + DeadCat windshield	6.0	5.6	−77.3
RØDE NTG-2 + parabolic dish	5.3	5.2	−79.3
Behringer B-1	5.1	4.5	−81.7
Behringer B-2	5.5	4.0	−78.0
Mozos VMic	5.4	1.2	−66.7

**Table 9:** DJI Avata 2—microphone performance during hover (mean over 1–50 m).

Microphone	Harmonic SNR [dB]	Wideband SNR [dB]	LF [dB]
RØDE NTG-2	17.3	7.6	−75.8
RØDE NTG-2 + WS6 windshield	16.8	12.0	−77.3
RØDE NTG-2 + DeadCat windshield	16.6	10.8	−76.8
RØDE NTG-2 + parabolic dish	17.6	9.8	−80.8
Behringer B-1	17.7	10.1	−84.8
Behringer B-2	15.3	7.9	−81.5
Mozos VMic	11.5	6.1	−66.6

**Table 10:** DJI Neo—microphone performance during hover (mean over 1–50 m).

Microphone	Harmonic SNR [dB]	Wideband SNR [dB]	LF [dB]
RØDE NTG-2	18.5	6.8	−72.1
RØDE NTG-2 + WS6 windshield	21.1	9.3	−75.2
RØDE NTG-2 + DeadCat windshield	20.7	8.8	−74.2
RØDE NTG-2 + parabolic dish	21.7	10.9	−76.3
Behringer B-1	19.0	7.0	−77.5
Behringer B-2	16.1	4.6	−73.8
Mozos VMic	12.5	3.6	−64.7

## Data Availability

The date presented in this study are available on request from the corresponding author.

## Abbreviations

The following abbreviations are used in this manuscript:

UAV	Unmanned Aerial Vehicle
DSP	Digital Signal Processing
SPL	Sound Pressure Level
SNR	Signal-to-Noise Ratio
RPM	Revolutions Per Minute
AGL	Above Ground Level
AI	Artificial Intelligence
CNN	Convolutional Neural Network
LSTM	Long Short-Term Memory
BPF	Blade Passage Frequency
BVLOS	Beyond Visual Line of Sight
RF	Radio Frequency
dBFS	Decibels Relative to Full Scale
PCM	Pulse Code Modulation
PSD	Power Spectral Density
LF	Low-Frequency
TDOA	Time Difference Of Arrival
SRP-PHAT	Steered Response Power with Phase Transform
MUSIC	Music Signal Classification
DOA	Direction of Arrival
